# ONRAMP-AI-VRAR: an operational protocol for ethics and governance of AI-enabled immersive psychotherapy

**DOI:** 10.1186/s12888-025-07557-x

**Published:** 2025-12-11

**Authors:** Aneesh Rahangdale, Jordan Schildkraut, Christopher Wadsworth

**Affiliations:** 1 HCA (Hospital Corporation of America), Florida Capital Hospital, University of Central Florida, 2626 Capital Medical Blvd, Tallahassee, FL 32308 USA; 2 Columbia Law School, New York City, NY USA; 3 University of California San Francisco, San Francisco, California USA

**Keywords:** Artificial intelligence ethics, Virtual reality (VR), Augmented reality (AR), Psychotherapy, ONRAMP, Neuroethics, Immersive technology, Risk mitigation, Cultural sensitivity, Regulatory frameworks

## Abstract

**Background:**

Artificial intelligence (AI)–powered virtual reality (VR) and augmented reality (AR) therapies offer promising tools for treating mental health conditions. However, their immersive and data-intensive nature introduces novel ethical, legal, and cultural risks not adequately addressed by existing frameworks.

**Objective:**

To introduce a structured ethical framework—Operational Neuroethical Risk Assessment and Mitigation Protocol for AI in Virtual and Augmented Reality (ONRAMP-AI-VRAR)—for identifying, mitigating, and governing risks specific to immersive AI-driven psychotherapy.

**Methods:**

We conducted a targeted literature review across PubMed, Scopus, PsycINFO, and the Cochrane Library using Boolean combinations of XR, mental health, ethics/risk, and AI terms. From an initial pool of 913 records, 15 studies met inclusion criteria (ethical principles, governance, or mitigation strategies relevant to XR psychotherapy). Following peer review, 3 additional conceptually relevant works were incorporated (e.g., Beg and Verma) into the narrative synthesis (total *n* = 18). Findings were coded to research questions (ethical, governance, implementation) and mapped to ONRAMP-AI-VRAR’s steps/tools.

**Results:**

ONRAMP-AI-VRAR translates recurrent risks into practical processes and artifacts: (1) a Biometric Bias Audit Checklist, (2) a Cultural Sensitivity Rubric for immersive content, (3) a Risk Prioritization Matrix, and (4) a VR/AR Data Flow Mapping Template for transparency and compliance. The protocol embeds human-in-the-loop oversight through a Multidisciplinary Oversight Board and proposes a tiered implementation model (Full, Integrated, Practitioner-level) to enhance feasibility across settings. It aligns with international guidance, including the EU AI Act, GDPR, WHO/UNESCO ethics recommendations, OECD AI Principles, and the NIST AI RMF.

**Conclusions:**

ONRAMP-AI-VRAR offers an actionable pathway to ethically deploy AI-enhanced immersive psychotherapy by integrating upfront audits, culturally aware design, layered consent, and continuous governance. The included tools are conceptual prototypes requiring psychometric validation. Future work should evaluate reliability, validity, and clinical impact across diverse populations and platforms.

**Clinical trial number:**

Not applicable.

**Supplementary Information:**

The online version contains supplementary material available at 10.1186/s12888-025-07557-x.

## Introduction

Artificial intelligence (AI)-enabled immersive technologies are rapidly reshaping mental health care. Virtual reality (VR) and augmented reality (AR), which may be referred to collectively as extended reality (XR), powered by adaptive AI systems have been developed to personalize psychotherapy, simulate controlled exposure environments, and deliver scalable interventions for anxiety disorders, post-traumatic stress disorder (PTSD), obsessive-compulsive disorder (OCD), attention-deficit/hyperactivity disorder (ADHD), and other mental health conditions. These tools aim to improve engagement, expand access to care in underserved areas, and reduce stigma by facilitating at-home or hybrid treatment models. However, their integration into clinical settings introduces novel ethical, legal, and social risks that existing frameworks are ill-equipped to manage.

Immersive mental health technologies differ from traditional digital therapies in their sensory intensity, real-time data capture, and capacity for autonomous adaptation. These features create new challenges surrounding autonomy, data privacy, cultural sensitivity, and algorithmic bias. For example, AI-driven exposure therapy may inadvertently overexpose users to triggering content or fail to adapt to individual psychological profiles, especially in populations underrepresented in training datasets [1]. Moreover, immersive technologies may increase the risk of user dissociation, privacy violations from biometric tracking, and re-traumatization without proper clinical oversight [2, 3]. Recent reviews highlight that while AI shows promise across a range of mental health applications, including ADHD, OCD, schizophrenia, and substance use disorders, there remains a lack of consensus on how to evaluate and implement these tools safely and ethically, particularly in immersive formats, underscoring the need for structured ethical guidance [4, 5].

To guide ethical and safe innovation, existing ethical frameworks such as the Belmont Report, the Health Insurance Portability and Accountability Act (HIPAA), and IRB-based research ethics offer general guidance but lack actionable tools tailored to the complexities of immersive AI systems. The Operational Neuroethical Risk Assessment and Mitigation Protocol (ONRAMP), first proposed by James Giordano [6], emphasizes the proactive identification of neuroethical, legal, and social issues (NELSI) across the lifecycle of neurotechnology development and deployment. However, ONRAMP has not yet been adapted into a concrete, implementable system for evaluating AI-integrated immersive therapies in mental health contexts. Responsible AI integration into mental health research calls for structured frameworks to integrate ethics with clinical practice [7].

To address this gap, we introduce the Operational Neuroethical Risk Assessment and Mitigation Protocol for Artificial Intelligence in Virtual and Augmented Reality (ONRAMP-AI-VRAR), a structured, six-step protocol specifically designed to assess and mitigate the unique risks of AI-powered immersive mental health technologies. This protocol builds on ONRAMP’s conceptual foundations by offering concrete tools and governance mechanisms tailored to immersive care, including a Biometric Bias Audit Checklist, a Cultural Sensitivity Rubric for immersive content, a Risk Prioritization Matrix for immersion-specific harms, and a Data Flow Mapping Template to promote transparency and regulatory compliance (Appendix A).

Immersive AI psychotherapy can be used across clinical environments, including therapists’ offices, hospitals, telehealth platforms, and even patient homes, especially where traditional care is inaccessible. However, to support innovation that centers patient dignity, cultural relevance, and psychological safety and prevent exacerbating disparities or causing unintended harm, transparent consent procedures, culturally informed content design, rigorous oversight mechanisms, and essential stakeholder engagement including therapists, patients, developers, ethicists, and regulators must be implemented. We foreground the “Garbage In, Garbage Out (GIGO)” problem for biometric and behavioral inputs: biased or low-fidelity data can propagate through adaptive pipelines to shape immersion, content selection, and clinical recommendations. ONRAMP-AI-VRAR embeds GIGO checkpoints during Scoping & Orientation (Step 1) and Structured Risk Identification (Step 3) to enforce data quality and representativeness before deployment.

## Methods

**Scope and Approach:** To inform development of the ONRAMP-AI-VRAR protocol, we conducted a targeted literature review of ethical, legal, and clinical risks associated with extended reality (XR), encompassing virtual and augmented reality (VR and AR) psychotherapy and AI-adaptive features. Our aim was not a comprehensive systematic review, but to ground the protocol in the most pertinent scholarship and guidance. Findings informing ONRAMP are synthesized in Table 1. In line with PRISMA 2020 guidance, we include an applicability-annotated checklist and a simple literature identification flow for transparency; no meta-analysis or formal risk-of-bias synthesis was planned or conducted. The review was not preregistered.

**Table 1:** Comparative review of XR/AI psychotherapy studies and gaps addressed by ONRAMP-AI-VRAR

Study/Source	XR/AI Application	Ethical/GIGO Risk Addressed	Tool/Framework Proposed	Gap Addressed by ONRAMP-AI-VRAR
Freeman et al. [8]	VR automated therapy for acrophobia	Minimal therapist input, risk of depersonalization	RCT efficacy outcomes only	**Step 4: Consent & Mitigation Planning; Tool 3: Risk Prioritization Matrix** (automation boundaries + consent scaffolds)
Falconer et al. [9]	VR embodiment for self-compassion	Risk of immersive over-identification	Experimental protocol only	**Step 4: Consent & Mitigation Planning; Tool 3: Risk Prioritization Matrix** (immersion-harm thresholds + pause/escape)
Mamede [10]	VR mental health applications	Privacy, consent, data security	Narrative overview	**Step 4: Consent & Mitigation Planning; Tool 4: VR/AR Data Flow Mapping Template** (layered/interactive consent + data transparency)
Jacobs [11]	AI-HCI in psychotherapy	Extended mind, privacy, autonomy	Conceptual essay	**Step 5: Implementation & Oversight** (tiered governance, MOB roles); **Step 4: Consent & Mitigation Planning** (consent boundaries for adaptive systems)
Warrier [12]	Metaverse + mental health	Algorithmic bias, equity	Ethical critique	**Step 3: Structured Risk Identification; Tool 1: Biometric Bias Audit Checklist** (subgroup performance, fairness docs)
Miller [13]	Apps, avatars, robots in MH	Algorithmic bias, cultural inclusivity	Perspective article	**Step 3: Structured Risk Identification; Tool 2: Cultural Sensitivity Rubric** (avatar/content review, inclusive design)
Dwivedi [14]	Negative societal impacts of metaverse	Safety, manipulation, governance	Theoretical analysis	**Step 5: Implementation & Oversight; Tool 3: Risk Prioritization Matrix** (governance triggers, incident response)
Njoh [15]	Dance movement therapy in XR	Equity, cultural representation	Narrative reflection	**Step 3: Structured Risk Identification; Tool 2: Cultural Sensitivity Rubric** (community review, language/imagery checks)
Schweizer [16]	VR trauma analogue	Patient safety (cybersickness, dissociation)	Experimental study	**Step 3: Structured Risk Identification; Tool 3: Risk Prioritization Matrix** (screening + exposure/intensity limits)
Morgan et al. [17]	VR cognitive therapy for depression	Bias in affective AI	None (exploratory trial)	**Step 3: Structured Risk Identification; Tool 1: Biometric Bias Audit Checklist** (affective model subgroup audit)
Hirzle et al. [18]	Ethical design framework for XR	Privacy, embodiment, autonomy	Ethical scaffolding model	**Step 4: Consent & Mitigation Planning; Tool 4: VR/AR Data Flow Mapping Template** (biometric/sensor consent); **Step 5: Implementation & Oversight** (governance scaffolds)
Pons et al. [19]	Review of immersive psychotherapy	Autonomy, immersion effects	Narrative synthesis only	**Step 4: Consent & Mitigation Planning; Tool 3: Risk Prioritization Matrix** (harm ladders, stop rules)
Spytzsa et al. [20]	AI-assisted VR trauma reprocessing	Suggestibility, patient agency	Conceptual risk typology	**Step 5: Implementation & Oversight** (role separation: AI vs clinician; escalation protocols) **+ Step 4** (human-in-the-loop)
Kourtesis et al. [21]	Psychometric VR assessment	Simulator sickness, validity	Measurement & protocol checklist	**Step 1: Scoping & Orientation** (psychometric anchors/GIGO) **+ Step 3: Tool 3** (risk scoring for sickness/validity)
Cox et al. [22]	Large-scale AI deployment	Consent complexity, scalability	Policy/system architecture suggestions	**Step 5: Implementation & Oversight** (MOB + quarterly audits) **+ Step 4: Tool 4** (data lineage/traceability)
[4]eg & Verma [7]	AI psychotherapy reviews	Governance, personalization, usability	Narrative reviews	**Step 3: Structured Risk Identification; Tool 1** (bias audit) **& Tool 2** (cultural rubric); **Step 4** (consent innovations); **Step 5** (tiered oversight)

This table summarizes the 15 studies and 3 AI psychotherapy reviews recommended by peer reviewers included in the targeted review (total *n* = 18), focusing on the ethical or GIGO (Garbage In, Garbage Out) risks addressed, whether a concrete tool or framework was proposed, and how ONRAMP-AI-VRAR fills identified implementation gaps. Each gap is mapped to a specific ONRAMP step and tool (see Appendix A for more details)

**Objectives:** The review sought to: (1) identify ethical risks and mitigation strategies specific to AI-enabled XR psychotherapy; (2) synthesize policies and governance models applicable to immersive/AI systems; and (3) extract implementation requirements that can be operationalized within ONRAMP steps/tools.

### Research Questions (RQs)

- **Ethical issues (RQ-E):** What immersion-/AI-specific risks (e.g., autonomy erosion, re-traumatization, cybersickness, privacy harms, algorithmic bias) are reported? Which mitigation strategies are proposed and how actionable are they (checklists, metrics, thresholds, consent innovations)? Where do tensions persist (scalability vs therapeutic alliance; autonomy vs adaptation; personalization vs standardization; privacy vs clinical utility)?
- **Policies/governance (RQ-G):** What governance models (IRB adjuncts, multidisciplinary oversight boards, tiered oversight) are described for AI/XR mental health? Which regulatory frameworks (HIPAA/GDPR, FDA SaMD, EU AI Act, NIST AI RMF) are referenced and with what concrete compliance expectations? What audit/transparency practices (data-flow mapping, change control, fairness documentation, logging) are recommended?
- **Clinical/implementation (RQ-C):** In what clinical contexts (diagnoses, populations, settings) has XR/AI psychotherapy been studied, and what risks/safeguards are context-dependent? What tools/measures (bias audits, cultural rubrics, risk matrices, psychometric anchors) could be embedded into ONRAMP? What resource/feasibility constraints (workforce, cost, literacy, infrastructure) shape implementation?

**Eligibility Criteria/Article Characteristics (driven by the RQs):** Inclusion targeted XR psychotherapy (VR/AR/MR) and/or AI features (adaptive content, affective computing, automated guidance) with relevance to ethics, risk, policy, or governance. Eligible sources included empirical studies reporting risks/harms or safeguards; conceptual/narrative works proposing governance, policy, or design frameworks; and reviews identifying implementation gaps. Exclusions: XR uses without mental-health relevance; engineering papers lacking ethical/governance content; purely technical works without clinical or policy implications (Tables 2–4).

**Table 2:** Ethical risk assessment tools

Tool	Name	Purpose
Tool 1	Biometric Bias Audit Checklist	Evaluate demographic fairness of biometric models. Assess training data diversity, sensor performance across demographic groups, and potential inequities in AI outcomes.
Tool 2	Cultural Sensitivity Rubric	Assess inclusivity, cultural appropriateness, and safety of therapeutic content, avatars, language, and framing.
Tool 3	Risk Prioritization Matrix	Prioritize mitigation for immersion-related harms. Rate harms by likelihood and impact, with categories for simulation sickness, re-traumatization, privacy breaches, etc. Outputs a Risk Prioritization Score to rank mitigations (no claim of clinical measurement precision).
Tool 4	VR/AR Data Flow Mapping Template	Map data usage for transparency and regulatory alignment. Document what data is collected (e.g., gaze, heart rate), how it is processed and stored, and who has access.

To operationalize ethical oversight, ONRAMP-AI-VRAR includes four structured tools for implementation and auditability. *Full tool templates are provided in Appendix* A.

**Table 3:** Recommended MOB membership

Role	Expertise & Function
Clinical Psychologist or Psychiatrist	Evaluate therapeutic integrity, screen for clinical risks
VR/AR Software Developer	Inform on system architecture, biometric sensors, and technical feasibility
Bioethicist or Neuroethicist	Weigh risk-benefit tradeoffs, autonomy, and justice
Data Privacy & Health Law Expert	Ensure compliance with HIPAA, GDPR, CCPA, and international AI governance
Patient Advocate/Peer Specialist	Center lived experience, accessibility, and community concerns

The MOB should include these members to support balanced decision making

**Table 4:** Legal foundations and ONRAMP-AI-VRAR safeguards for AI in immersive psychotherapy

Legal Doctrine/Statute	Key Legal Case or Statute (doctrine)	AI Healthcare Application	ONRAMP-AI-VRAR Safeguard
Malpractice & Duty of Care	**Hawkins v. McGee (1929)** [23] *(Contract/Expectation Damages; representations to patients)*	Overreliance on AI therapy recommendations or outcome promises	**Human-in-the-loop** clinical accountability; document limits of AI recommendations; avoid promising outcomes derived from AI outputs; clinician sign-off recorded in audit trail.
Duty to Protect/Duty to Warn (Tarasoff)	**Tarasoff v. Regents of the Univ. of California (1976)** [24] *(Duty to protect/warn identifiable third parties)*	AI-assisted detection of harm to others or self	**MOB-approved escalation thresholds**; real-time risk flags to clinicians; documented outreach pathways and timelines; post-incident review and remediation.
Institutional Responsibility	**Darling v. Charleston Community Memorial Hosp. (1965) **[25]*(Corporate negligence/institutional liability)*	AI integration into hospital/clinic workflows	**Standing governance** (MOB or integrated committee); competency and credentialing for systems and staff; **system-level audit trails** and periodic safety/equity audits.
FDA Preemption	**Riegel v. Medtronic, Inc. (2008)** [26]*(Preemption of certain state claims for FDA-approved devices)*	FDA-regulated **SaMD** (including adaptive XR/AI modules)	Correct **SaMD pathway** selection; change-control and traceability logs for model/content updates; post-market surveillance and adverse-event reporting.
Statutes of Limitation	**Jurisdiction-dependent** *(Discovery rule/accrual timing)*	Latent or cumulative AI-related harms discovered later	**Longitudinal logging** (versions, timestamps, events); provenance for data/models; preserved audit trails to establish chronology and accountability.
Transparency of AI Use	**California AB 3030 (2024) **[27] *(Disclosure of AI-generated patient communications)*	Patient-facing generative outputs or automated messages	**Layered disclosure** in consent and UI labels when AI content is shown; user-viewable message provenance; periodic re-consent after material system updates.
Human Clinical Judgment	**California SB 1120 (2024)** [28] *(“Physicians Make Decisions Act”: no AI-only determinations)*	Coverage/treatment determinations or high-stakes clinical actions	**Explicit human validation gates** for high-risk decisions; recorded clinician attestation; blocks on AI-only determinations in workflows.

This table summarizes key legal doctrines, statutes, and landmark cases relevant to AI applications in mental health care. For each legal precedent, the corresponding risk scenario in AI-driven immersive therapy is described alongside the ONRAMP-AI-VRAR protocol safeguard designed to address the issue

**Search Strategy:** We searched PubMed, Scopus, PsycINFO, and the Cochrane Library from database inception to 08/2025 using combinations of terms for immersive technology (“virtual reality,” “augmented reality,” “mixed reality,” “extended reality,” “XR”), mental health applications (“psychotherapy,” “cognitive behavioral therapy,” “counseling,” “mental health,” “psychiatry”), ethics/risk concepts (“ethics,” “risk assessment,” “bias,” “privacy,” “informed consent,” “governance,” “policy,” “neuroethics”), and adaptive systems (“artificial intelligence,” “machine learning,” “adaptive systems”), supplemented by citation chasing and hand-searching key authors/outlets. Search strings were adapted per database. Non-English items and engineering-only reports without clinical relevance were excluded at screening.

**Screening and Selection:** Titles/abstracts were screened for relevance to AI-enabled VR/AR psychotherapy and ethics/governance to exclude clearly irrelevant use cases (e.g., physical/occupational/speech/sports therapy; engineering without clinical application). Potentially eligible reports underwent full-text review. Full texts were included if they addressed risks, ethical principles, regulatory issues, or mitigation strategies relevant to immersive technologies in psychotherapy or mental health care. Studies describing VR/AR interventions without reference to ethics or risk were excluded. Reasons for exclusion at full text (e.g., not psychotherapy, engineering-only, no ethics/governance) are summarized in the PRISMA flow. Data were extracted into structured domains (intervention context, risks/harms, governance elements, mitigation strategies) and mapped to ONRAMP steps and tools; no quantitative pooling was undertaken.

**Coding, Synthesis, and Mapping:** From 913 records, 15 studies met inclusion criteria. Following peer review, 3 conceptually relevant works (e.g., Beg; Verma) were added to the narrative synthesis (total = 18). Eligible articles were coded to RQ domains (Ethical; Governance/Policy; Clinical/Implementation) and mapped to ONRAMP by: (1) aligning themes with Steps 1–6 (Scoping & Orientation; Gap & Needs Assessment; Structured Risk Identification; Consent & Mitigation Planning; Implementation & Oversight; Review & Iteration); (2) linking concrete recommendations to Tools 1–4 (Biometric Bias Audit Checklist; Cultural Sensitivity Rubric; Risk Prioritization Matrix; VR/AR Data Flow Mapping Template); and (3) capturing trade-offs/tensions to inform governance (e.g., boundaries for human-in-the-loop vs automation). This ensured each protocol element is explicitly justified by the literature and tied to specific RQs. We used narrative synthesis: concept clustering and matrix mapping to ONRAMP steps (risk ID, consent/mitigation, oversight tiering, competencies). Where applicable, scales are defined at first mention (e.g., PTSD Checklist for DSM-5 (PCL-5), Panic Disorder Severity Scale–Self-Report (PDSS-SR), Dissociative Experiences Scale–II (DES-II)).

## Background: XR/AI psychotherapy and ethical gaps

From the 18 relevant studies in the targeted literature review, we identified several recurring ethical themes. The most frequently cited concerns were risks to privacy and data security, particularly around biometric and behavioral information captured by immersive devices [10, 11]. Informed consent in adaptive VR/AR environments was also consistently highlighted, often emphasizing the need for enhanced disclosure mechanisms [10, 11]. Multiple papers noted risks of algorithmic bias in AI-driven immersive systems [12, 13]. Concerns related to patient safety, including cybersickness, dissociation, and misuse of VR technologies, were also identified [14, 16]. A smaller number of studies called for comprehensive ethical frameworks and raised issues of equity and cultural sensitivity, particularly in relation to access and inclusivity [10, 15]. Mentions of governance and regulatory oversight were rare, though some conceptual analyses underscored the need for clearer policy and system-level safeguards [14, 18].

Alongside these ethical analyses, empirical evidence has demonstrated the clinical promise of immersive therapy. Freeman et al. (2017) [8] conducted a landmark randomized controlled trial (RCT) of automated VR therapy for acrophobia, demonstrating significant reductions in fear levels with minimal therapist input. Similarly, Falconer et al. (2016) [9] found that VR environments designed to cultivate self-compassion reduced depressive symptoms, highlighting the potential of embodiment-based interventions. Embodiment-based interventions are therapies that change how a person experiences their body or point of view to produce psychological benefits. In XR/VR, that usually means using avatars, first-person perspective, and synchronized sensory cues. Systematic reviews have further supported these findings: Kourtesis et al. [21] emphasized strong efficacy signals across psychiatric diagnoses while cautioning against simulator sickness and overstimulation, and Cox et al. [22] stressed the importance of inclusive design and representative training datasets for AI-enhanced XR platforms. Other applied studies have explored specific clinical use cases, such as Morgan et al. [17] on depression-focused XR therapy and Pons et al. [19] on youth anxiety, while also pointing to limitations in tailoring affective AI responses and risks of re-traumatization in unsupervised contexts.

Ethical scholarship in this area has advanced toward design-level frameworks. Hirzle et al. [18] proposed a design-centered ethical model addressing autonomy, embodiment, and consent, noting how real-time biometric tracking can blur boundaries between therapeutic intervention and behavioral manipulation. They highlighted the risks of reduced user agency, dissociation, and unintended psychological influence in vulnerable populations. Spytzsa et al. [20] extended this work by developing a taxonomy of ethical risks for AI-assisted psychotherapy, categorizing them by data origin, algorithmic function, and decision-making control. Their work underscores the need for transparency and modularity in therapeutic AI, ensuring that system components (e.g., emotion detection, content selection, session guidance) remain explainable and clinically accountable.

Finally, several narrative reviews, brought to our attention by courtesy of our peer reviewers, have sought to situate XR- and AI-based psychotherapy within broader ethical and clinical discourses. Beg and Verma [4] emphasize the importance of culturally embedded and user-centered design, noting that personalization, usability, and contextual relevance are critical for engagement and therapeutic impact in digital and AI-driven interventions for conditions such as ADHD, OCD, and schizophrenia. These authors also highlight the need for informed consent innovations that move beyond static disclosure forms toward interactive, adaptive consent processes aligned with the dynamic nature of AI systems. In a related review, Beg et al. [5] survey AI applications in psychotherapy, underscoring both their promise, such as chatbot-mediated cognitive behavioral therapy for depression and anxiety, and their limitations, particularly with respect to trust, privacy, and the preservation of the therapeutic alliance. They argue that conversational agents represent a critical frontier where immersive environments and therapeutic AI converge, yet also caution that issues of crisis management, emotional reciprocity, and cultural sensitivity remain unresolved. Complementing these perspectives, Beg [7] outlines principles of responsible AI integration in mental health research, calling for transparency, accountability, bias mitigation, and rigorous human oversight to ensure that AI augments rather than substitutes clinical expertise.

Despite a growing body of commentary on XR- and AI-enabled psychotherapy, persistent ethical gaps remain, reflecting both structural limitations and unresolved normative disagreements. At a structural level, oversight frameworks are fragmented across jurisdictions and disciplines, leaving little coordinated guidance for clinicians. The rapid pace of technology development routinely outstrips the ability of regulators and ethicists to respond, while most published work originates in high-income settings, limiting cultural applicability and reinforcing inequities. Equally important, very few of the ethical principles articulated in the literature have been empirically validated in clinical contexts, leaving uncertainty about how they function in practice. Against this backdrop, the literature itself reflects sharp tensions. Optimists highlight scalability, personalization, and enhanced engagement, while critics warn of erosion of therapeutic alliance and increased depersonalization. Some frame adaptive systems as expanding patient autonomy, whereas others see automation as constraining meaningful choice. Similarly, personalization of immersive content promises more precise tailoring, yet raises concerns about reproducibility, safety testing, and equitable access. Finally, unresolved debates around data collection pit clinical utility against privacy, as the same biometric and affective data that enable responsive therapy also expand the potential for surveillance and misuse. These combined structural barriers and substantive disagreements underscore that ethical “gaps” in XR-based psychotherapy are not simply the result of oversight failures but reflect deeper conflicts of values and priorities within the field.

Addressing these challenges requires a framework that is both pragmatic and principled. ONRAMP-AI-VRAR was designed with this dual mandate: to close structural gaps in oversight by offering a stepwise protocol and practical tools that can be adapted across settings, while also making explicit the trade-offs embedded in current debates. By linking identified gaps to specific ONRAMP steps and tools (for example, using the Biometric Bias Audit Checklist to confront inequities or the Risk Prioritization Matrix to balance personalization with safety) ONRAMP moves beyond descriptive commentary to provide an actionable roadmap. In doing so, it aims not to resolve all disagreements, but to surface them in a structured manner that allows clinicians, researchers, and policymakers to navigate contested priorities with transparency and accountability. Table 1 maps these findings to ONRAMP-AI-VRAR’s risk domains, underscoring how the proposed protocol builds upon and extends prior work by introducing implementation-focused tools and safeguards (Fig. 1).

## The ONRAMP-AI-VRAR protocol

**Fig. 1:**
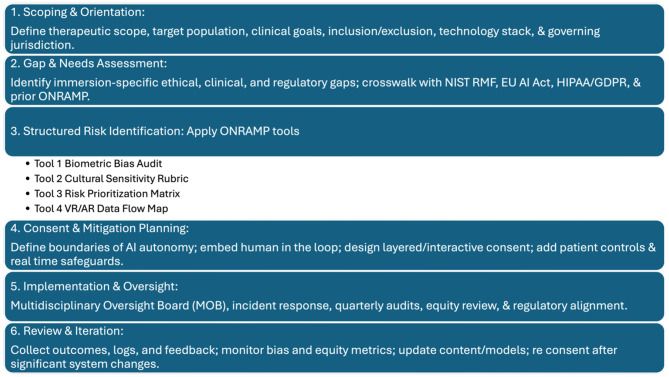
The operational neuroethical risk assessment and mitigation protocol for artificial intelligence in virtual and augmented reality (ONRAMP-AI-VRAR) is a structured, six-step framework for scoping, risk identification, consent/mitigation planning, implementation with oversight, and continuous review for AI-enabled immersive psychotherapy. Adapted from Giordano’s ONRAMP model [6], this protocol translates abstract ethical guidance into practical processes and tools. Included tools: (1) biometric bias audit, (2) cultural sensitivity rubric, (3) risk prioritization matrix, (4) VR/AR data flow mapping. It is aligned with international standards such as the European Union (EU) Artificial Intelligence act (EU AI Act), the General Data Protection Regulation (GDPR), the National Institute of Standards and Technology (NIST) Artificial Intelligence Risk Management Framework (AI RMF), and ethics guidance from the United Nations Educational, Scientific, and Cultural Organization (UNESCO) and World Health Organization (WHO). ONRAMP-AI-VRAR is intended for clinicians, developers, institutional review boards (IRBs), and multidisciplinary oversight boards (MOBs) involved in the deployment of immersive psychotherapy. It addresses risks related to biometric data, immersion intensity, algorithmic bias, consent, cultural sensitivity, and regulatory compliance

### Step 1: Scoping & orientation

Define the scope of the intervention and its technological, clinical, and regulatory context.

- Identify the target population (e.g., PTSD in veterans, social anxiety in adolescents)
- Clarify clinical goals and inclusion/exclusion criteria
- Describe the hardware/software stack (e.g., sensors, adaptive AI, XR platforms)
- Declare the relevant legal jurisdiction (e.g., U.S., EU, hybrid)

### Step 2: Gap & needs assessment

Map potential ethical and clinical risks specific to immersive, AI-powered therapies.

- Conduct a literature review and stakeholder interviews to identify novel psychological harms (e.g., derealization, dissociation, re-traumatization), immersion-specific issues (e.g., sensory overload, spatial privacy, sensor misuse), and AI-specific risks (e.g., opaque decision-making, algorithmic bias, autonomy erosion)
- Crosswalk these risks with existing frameworks (ONRAMP, NIST RMF, EU AI Act) to highlight gaps

### Step 3: Structured risk identification

Apply structured tools to identify and evaluate risks (see Appendix A for detailed tools).

### Step 4: Consent & mitigation planning

Develop strategies to proactively reduce or eliminate identified risks.

- Define clear boundaries for AI autonomy (e.g., what actions require human validation)
- Embed human-in-the-loop features (e.g., dashboards for therapists to monitor distress signals in real-time)
- Design layered consent procedures (e.g., interactive demos, tiered disclosure levels)
- Integrate patient-controlled safety features (e.g., pause triggers, emergency handoff protocols)
- Define exposure intensity ceilings and panic-action plans in the care plan; require human validation before advancing beyond preapproved exposure steps for patients with high-risk stratifiers
- Document **example stop-rules** (any → pause/terminate and de-escalate): sustained subjective distress ≥7/10 for > 60 s; emergence of dissociative phenomena (e.g., marked derealization or **DES-II** red-flag items); notable cybersickness (moderate N/V or vertigo); or clinician-judged autonomic instability (e.g., hyperventilation, presyncope).
- Include patient-facing “how to stop” language in consent and display an always-visible pause/exit control in the interface.

### Step 5: Implementation & oversight

Establish ongoing human oversight and governance (see “Oversight Governance” section for more details).

- Form a Multidisciplinary Oversight Board (MOB) with at least:
  o A clinical psychologist or psychiatrist
  o An XR/AI systems developer
  o A bioethicist
  o A legal/data privacy expert
  o A patient or caregiver representative
- MOB responsibilities include:
  o Approving mitigation strategies and deployment plans
  o Reviewing incident reports and adverse events
  o Conducting quarterly reviews of equity impacts, content updates, and data safety audits

### Step 6: Review & iteration

Embed continuous evaluation and adaptive updates.

- Collect patient-reported outcomes, system logs, biometric trends, and clinician feedback
- Flag discrepancies between AI and clinician recommendations for human review
- Update protocols based on new evidence, adverse events, bias detection, and evolving cultural concerns

This six-step protocol enables a repeatable, stakeholder-informed process to ensure immersive AI therapies prioritize safety, autonomy, equity, and transparency.

## Key ethical focus areas

This section integrates key ethical risks with corresponding protocol tools and mitigation strategies, focusing on autonomy, privacy, cultural sensitivity, AI boundaries, accessibility, psychological safety, and public trust.

### Patient autonomy and informed consent

Immersive and AI-driven therapy introduces novel risks to patient autonomy. Users may not fully comprehend how behavioral and biometric data (e.g., gaze tracking, facial expression, movement) are collected, interpreted, and used in therapy, especially given the complexity of these systems and their underlying algorithms [2].

ONRAMP-AI-VRAR’s Data Flow Mapping Template promotes transparency by documenting what data is captured and for what purpose, where and how long it is stored, and who has access (e.g., clinician, developer, system admin). Consent processes should include interactive VR walkthroughs, layered digital modules with progressive disclosures, and periodic re-consent after significant system updates. These strategies are particularly important for vulnerable populations (e.g., minors, individuals with trauma or neurodivergence) who may struggle with abstract or legalistic consent documents [29]. AI systems should also include customizable settings to honor patient preferences and allow individuals to opt out or modify their therapeutic exposure during care [2, 23].

### Privacy and data security

Immersive systems generate vast amounts of sensitive data, including physiological responses, affective cues, and spatial telemetry, that require rigorous safeguards. These datasets grow increasingly valuable and complex, escalating the ethical imperative to protect them from misuse or breaches [2].

Using ONRAMP-AI-VRAR’s Data Flow Mapping Template, systems must ensure encryption, anonymization, access controls and comply with HIPAA, GDPR, and CCPA if U.S.-based, or EU MDR/AI Act if international. Oversight boards should review storage protocols, access logs, and data retention policies regularly. In early-stage systems, multiple licensed professionals should review session content and datasets to detect algorithmic bias or emergent risk patterns [23].

### Algorithmic bias, fairness, and cultural sensitivity

AI systems trained on narrow datasets may reinforce real-world inequities, particularly in interpreting emotion, language tone, or physiological distress markers, due to demographic variation in these expressions [1, 30].

ONRAMP-AI-VRAR incorporates Biometric Bias Audit Checklists to test subgroup performance, synthetic demographic simulations to evaluate disparity risk, and transparent fairness metrics and documentation pipelines. The Cultural Sensitivity Rubric must be applied to immersive content to avoid harmful stereotypes, ensure inclusivity across cultural and demographic lines, and require review by diverse professionals and advocates (Appendix A). Even with robust safeguards, some bias may persist due to systemic data limitations, necessitating iterative peer review and adaptive model retraining [24].

### AI autonomy vs. human oversight

Striking the right balance between automation and clinical supervision is essential. Early iterations should include robust human-in-the-loop structures, particularly for high-risk decisions [31].

ONRAMP-AI-VRAR Step 4 requires clear delineation between AI-autonomous actions (e.g., modulating exposure length), human-mediated decisions (e.g., introducing trauma content), real-time dashboards for clinician review, and post-session logs for board oversight. Therapists must retain ultimate decision-making authority to prevent erosion of the therapeutic alliance and ensure clinical accountability [2].

### Accessibility and equity

Without proactive design, AI-enhanced immersive therapy could deepen mental health inequities. Barriers include cost, connectivity, spatial constraints, and low digital literacy, especially in low-income and rural populations [3]. The protocol recommends a few ideas: mobile-first and low-bandwidth design, Medicaid-funded subsidies and/or community distribution, deployment in libraries and community centers, and demographic audits during performance review cycles. These interventions aim to expand rather than restrict access to care [23].

### Psychological harms from immersion

Immersive interventions may induce simulation sickness, derealization, or psychological distress, particularly in higher acuity and complexity patients. Risk includes re-traumatization via virtual exposure, panic responses and dissociative episodes, and somatic symptoms like nausea or vertigo. Risk is a function of patient factors, content intensity, and autonomy level of the system rather than the modality itself. ONRAMP-AI-VRAR operationalizes this with pre-session screening (for example, PTSD Checklist for DSM-5 (PCL-5), Panic Disorder Severity Scale–Self-Report (PDSS-SR), and Dissociative Experiences Scale–II (DES-II)), graded exposure ladders, real-time distress detection with patient-controlled pause, predefined stop-rules, and rapid escalation pathways to a human clinician, with tier escalation when high-risk stratifiers are present [3].

### Public trust and ethical transparency

Public acceptance of AI psychotherapy depends on transparency and demystification. Tools like published audit logs, open-access performance dashboards, and oversight board summaries build public legitimacy and inform users, clinicians, and regulators [32]. Educational campaigns can reframe AI as an extension of human care. These campaigns may also reduce stigma by offering more discreet, scalable entry points for patients hesitant to seek traditional therapy [2, 26]. Moreover, alignment with global frameworks, such as the NIST AI RMF, WHO’s Guidance on Ethics and AI, and UNESCO’s global AI ethics recommendations, ensures international relevance and consistency [6].

## Oversight governance

### The roles of the multidisciplinary oversight board (MOB)

As outlined in Step 5 of the ONRAMP-AI-VRAR protocol, all immersive AI psychotherapy deployments must be subject to review and continuous monitoring by a Multidisciplinary Oversight Board (MOB) that meets ideally quarterly. This structure is essential to operationalize neuroethical governance and ensure ethical, legal, cultural, and clinical accountability throughout the technology’s lifecycle [6, 26]. The MOB approves needs assessments, risk identification, and mitigation plans (ONRAMP-AI-VRAR Steps 2–4), reviews immersive therapeutic content using the Cultural Sensitivity Rubric, oversees incident reporting systems, and investigates adverse psychological events. Further, the MOB conducts audits of algorithmic drift and fairness, consent fidelity and user comprehension, privacy compliance and data security, and disparities in clinical performance across demographic groups. Additionally, the MOB vets software updates and data pipeline changes for legal and ethical alignment [33], engages in scenario modeling to anticipate emergent ethical-legal conflicts [26], and publishes transparent, de-identified reports to maintain public trust. Regular risk assessments and feedback loops enhance adaptability to fast-evolving AI capabilities and user needs [25].

Rotating advisors may include cultural anthropologists, child and adolescent mental health specialists, disability rights experts, and AI fairness and transparency researchers to broaden involved stakeholders [1, 26].

Meeting frequency and triggers include initial approval review before first deployment of any therapeutic module, quarterly oversight meetings to review performance metrics, system logs, and user feedback, and ad hoc reviews triggered by major algorithm or software updates, flagged adverse events, and significant requests from patients or therapists. Therapist-facing platforms should include real-time flagging mechanisms for MOB escalation, and patient feedback collection modules and session audit logs should be accessible to board members under strict privacy controls. All decisions and audits should be recorded in a secure compliance system, with summaries released publicly to promote ethical transparency [23]. Ideally, MOB decisions are binding for participating institutions using the ONRAMP-AI-VRAR framework.

## Legal and regulatory frameworks (U.S. and Global)

ONRAMP-AI-VRAR aligns with the Health Insurance Portability and Accountability Act (HIPAA), the General Data Protection Regulation (GDPR), the U.S. Food and Drug Administration’s Software as a Medical Device framework (FDA SaMD), the Office of the National Coordinator for Health Information Technology’s Health IT Certification Program (ONC HTI-1), the European Union Artificial Intelligence Act (EU AI Act), the European Union Medical Device Regulation (EU MDR), the National Institute of Standards and Technology Risk Management Framework (NIST RMF), the World Health Organization (WHO), the United Nations Educational, Scientific and Cultural Organization (UNESCO), and the Organization for Economic Co-operation and Development (OECD) AI ethics guidelines. Implementation requirements include risk mapping, data governance, and global compliance.

### Framing the regulatory landscape: U.S. and global perspectives

The ONRAMP-AI-VRAR protocol is designed for broad applicability across jurisdictions. While much of its terminology and structure aligns with U.S. legal frameworks such as HIPAA and the FDA’s Software as a Medical Device (SaMD) guidance, the protocol also incorporates global ethical and legal standards. Developers and clinical adopters should explicitly identify whether the therapeutic system will be deployed under U.S.-specific legal frameworks (e.g., HIPAA, CCPA, FDA, ONC), European Union regulations (e.g., General Data Protection Regulation [GDPR], Medical Device Regulation [MDR], and the AI Act), and/or international ethical guidance (e.g., WHO, UNESCO, OECD).

### U.S.-based regulatory guidance

In the United States, the following bodies and frameworks guide AI-based therapeutic technologies:

- **HIPAA**: Ensures privacy and security of patient health information.
- **FDA SaMD Guidance**: Defines regulatory pathways for software-based medical interventions.
- **ONC HTI-1 Final Rule**: Requires algorithmic transparency and real-world performance disclosure for health IT tools.
- **NIST Risk Management Framework (RMF)**: Offers a modular, tiered framework for assessing and mitigating cybersecurity and AI risks in high-stakes domains, including healthcare (NIST, 2023).
- **FTC AI Transparency Guidelines**: Address AI explainability, fairness, and consumer protection.

### European and international standards

In the European Union:

- The **EU AI Act** designates most mental health AI applications as high-risk systems, requiring strict data governance, human-in-the-loop oversight, and traceability mechanisms [34].
- The **EU Medical Device Regulation (MDR)** governs therapeutic software and VR systems when used for clinical diagnosis or treatment [35].
- The **GDPR** sets global benchmarks for patient data access, portability, and erasure.

Globally relevant ethical standards include:

- **UNESCO Recommendation on the Ethics of Artificial Intelligence**: Emphasizes human rights, cultural diversity, and environmental sustainability [36].
- **WHO Guidance on Ethics and Governance of AI for Health**: Calls for inclusivity, explainability, and real-world testing prior to deployment (WHO, 2021).
- **OECD AI Principles**: Adopted by over 40 countries, these principles promote transparency, robustness, and accountability in trustworthy AI systems (OECD, 2019).

ONRAMP-AI-VRAR incorporates key elements of these frameworks by requiring cultural review of therapeutic content (UNESCO, WHO), embedding human oversight and review boards (EU AI Act, NIST), demanding data transparency and auditability (GDPR, FTC, ONC), and establishing patient-centered safeguards for vulnerable populations (OECD, WHO).

### Implications for compliance

To ensure ethical and legal compliance, institutions adopting immersive AI therapy must map the protocol to jurisdiction-specific requirements, implement layered consent aligned with GDPR and CCPA, submit SaMD tools for premarket review when required, and build traceability and explainability features to meet AI Act and NIST expectations. International deployment requires local legal consultation and engagement with regulatory authorities to ensure ONRAMP-AI-VRAR components (e.g., audit trails, oversight boards, cultural review) meet jurisdictional standards.

### U.S. Legal precedents and liability implications

While ONRAMP-AI-VRAR emphasizes global compliance and proactive ethical design, developers and deployers in the United States must also consider key liability doctrines and case law shaping clinical responsibility for AI-driven mental health interventions.

1. **Malpractice and Duty of Care**
  The traditional malpractice standard requires clinicians to act in accordance with professional norms. *Hawkins v. McGee* (1929) [23] affirmed that providers can be held accountable for outcomes they explicitly promise, a principle with implications for overreliance on AI recommendations. Although AI systems may inform decisions, ultimate responsibility remains with licensed professionals.
2. **Duty to Warn and Third-Party Liability**
  *Tarasoff v. Regents of the University of California* (1976) [24] established a duty for therapists to protect identifiable third parties from foreseeable harm. If AI systems detect risk and clinicians fail to act, or if systems fail to detect threats, liability may extend to developers and/or providers.
3. **Institutional Responsibility and Respondeat Superior**
  *Darling v. Charleston Community Memorial Hospital* (1965) [25] held hospitals liable for failing to ensure the competence of systems and staff. This principle may extend to AI-integrated workflows. When immersive AI tools are embedded in care processes, institutions may bear responsibility for oversight failures.
4. **FDA Preemption and AI Medical Devices**
  *Riegel v. Medtronic, Inc.* (2008) [25] held that FDA approval of a device may preempt certain state tort claims. This affects SaMD tools governed under ONRAMP-AI-VRAR, especially when adverse outcomes arise from malfunctioning or adaptive AI systems.
5. **Statutes of Limitation in AI Harm**
  Unlike discrete clinical interventions, AI-related harm may emerge over time. Legal debates persist regarding whether the limitation period begins at deployment, harm, or discovery. Institutions should maintain audit trails to support defensible timelines and traceability.

### Emerging statutory developments

Recent state-level legislation reflects growing concern over AI in healthcare:

- **California AB 3030 **[27]: Mandates disclosure when generative AI is used in patient-facing communication.
- **California SB 1120** [28] (“Physicians Make Decisions Act”): Prohibits insurance denials based solely on AI outputs and requires human clinical judgment for coverage.

These trends underscore the importance of governance models like the Multidisciplinary Oversight Board (MOB), enabling proactive monitoring, legal preparedness, and transparent escalation protocols.

## Risk identification and mitigation

Each immersive therapy risk is linked to a tool from ONRAMP and assigned a tailored mitigation strategy. See Table 5 for a synthesis of common risks and corresponding mitigation tools.

**Table 5:** ONRAMP-AI-VRAR risk identification and mitigation table

Risk Category	Specific Ethical or Clinical Risk	Tool/Protocol Element Used
Bias and Discrimination	Algorithmic unfairness from unrepresentative biometric training data	Tool 1: Biometric Bias Audit Checklist
Consent and Comprehension	Users unable to understand immersive data flows or risks using traditional consent formats	Step 1: Scoping & Orientation; Step 5: Implementation & Oversight; interactive consent + Tool 4: VR/AR Data Flow Mapping Template
Privacy and Surveillance	Invasive or unclear tracking of user behavior, speech, or physiological responses	Tool 4: VR/AR Data Flow Mapping Template
Algorithmic Drift	AI systems evolve without accountability, introducing new or undetected risks over time	Step 4: Consent & Mitigation Planning; Step 5: Implementation & Oversight (monitoring, change control, post-market review)
Psychological Harm	Dissociation, re-traumatization, or simulator sickness due to immersion	Tool 3: Risk Prioritization Matrix
Cultural Incongruence	Avatars, narratives, or symbols that perpetuate stereotypes or exclude minority users	Tool 2: Cultural Sensitivity Rubric
Overreliance on Automation	AI systems make critical clinical decisions without adequate human review	Step 5: Implementation & Oversight; Therapist Dashboard
Legal Non-Compliance	Failure to meet standards for AI/health data regulation (e.g., HIPAA, GDPR, FDA SaMD, EU AI Act)	Tool 4: VR/AR Data Flow Mapping Template

This table maps key ethical and clinical risks associated with immersive AI psychotherapy to specific steps in the ONRAMP-AI-VRAR protocol. Each risk is matched with structured tools or safeguards—such as bias audits, consent matrices, or oversight board mechanisms—to guide mitigation and ensure ethical compliance throughout the system lifecycle. For detailed mitigation plans and clinical implementation steps, see Appendix B

## Implementation & scalability

While the ONRAMP protocol was designed to provide comprehensive oversight, its feasibility varies across settings. The originally described model, quarterly meetings of a dedicated Multidisciplinary Oversight Board (MOB), is realistic only for large research institutions or well-funded commercial deployments. To improve scalability, we propose a tiered implementation model:

**Tier 1: Full Implementation** – The complete ONRAMP framework, including a standing MOB with quarterly reviews, is recommended for high-risk research studies or large-scale commercial products involving sensitive biometric data

**Tier 2: Integrated Implementation** – Hospitals and larger clinics may integrate ONRAMP oversight into existing structures such as Institutional Review Boards or clinical ethics committees, streamlining review while preserving safeguards.

**Tier 3: Practitioner-Level Implementation** – For smaller practices or early adopters, a lightweight version emphasizing use of the ONRAMP checklists and matrices within supervision or case formulation offers a pragmatic entry point.

### Tier assignment rubric (objective/quantitative)

To select an appropriate oversight tier, we propose a **Governance Tier Score (GTS)** that sums seven objective factors (0–3 each; higher = more governance need). Scores map to tiers: **Tier 1 ≥ 14**, **Tier 2 = 8–13**, **Tier 3 ≤ 7**.

**Table 6:** Tier assignment rubric

Factor (0–3)	0	1	2	3
Clinical Risk Level (acuity of target use)	Wellness/psychoeducation	Low-risk anxiety/exposure	Moderate risk; comorbidities	High-risk (e.g., suicidality, PTSD re-exposure)
Data Sensitivity (breadth/depth)	Minimal metadata only	PII/PHI (limited)	PHI + behavioral streams	PHI + biometric/affective + audio/video
Scale of Deployment (N active users/mo)	< 25	25–99	100–499	≥500/multi-site
Phase	Prototype/offline testing	Pilot (single site)	Clinical study (multi-arm)	Clinical service/commercial rollout
AI Autonomy Level	Decision support only	Suggests content	Modulates session flow	Autonomously selects/advances exposure
Competency Gap (staff vs. required skills)	Fully trained; DECODE-aligned	Minor gaps	Notable gaps; upskilling planned	Major gaps; external support required
Resources/Manpower (FTEs, budget)	Dedicated team and budget	Adequate	Tight	Insufficient without added support

The rubric quantitatively balances risk, data sensitivity, scale, phase, autonomy, competency, and capacity, the factors most determinative of oversight burden. It can be applied prospectively (pre-deployment) and iteratively (Step 6) to escalate/de-escalate oversight as conditions change. Governance Tier Score (GTS) = sum of the seven 0–3 ratings (range 0–21). Tier selection: Tier 1 (Full) if GTS ≥ 14; Tier 2 (Integrated) if 8–13; Tier 3 (Practitioner) if ≤ 7

### Clinical risk stratifiers

For the Governance-Tier Score clinical-risk item, “Moderate” and “High” risk should be assigned when any of the following are present: active suicidality or recent self-harm; psychotic features; bipolar I disorder with recent mania or mixed features; severe panic disorder with recent attacks; PTSD with prominent dissociation or high DES-II (dissociative experiences scale) score; borderline personality disorder with recent impulsive self-injury; active substance use disorder with recent withdrawal risk; seizure disorders or significant traumatic brain injury; severe vestibular or migraine syndromes that increase cybersickness susceptibility; unstable cardiovascular disease where autonomic stress may be hazardous; sensory-processing vulnerabilities that limit tolerability (for example, autism with sound or visual hypersensitivity). These stratifiers escalate oversight tier and invoke stricter stop-rules in the Risk Prioritization Matrix, with human-in-the-loop supervision required for exposure content.

### Oversight operations: practical guidance

#### Tier 1—Full implementation (Multidisciplinary oversight board/MOB)

**Composition (minimum):** psychiatry/psychology clinician; XR/AI engineer; data privacy/health law; bioethicist; patient/peer advocate.

**Cadence:** standing MOB; **quarterly** reviews; **ad hoc** for major updates/events.

##### Annual oversight plan (template)

- **Q1:** Approve risk register; verify **Tool 4** data-flow map; review consent UX; confirm safety thresholds/kill-switches.
- **Q2:** Equity audit (subgroup performance via **Tool 1**); cultural content review via **Tool 2**; update mitigation plan (**Step 4**).
- **Q3:** Security/privacy audit; incident drill (threat escalation per Tarasoff); post-market surveillance summary (if SaMD).

- **Q4:** Year-end Risk Prioritization Matrix refresh (Tool 3); policy alignment check (HIPAA/GDPR, EU AI Act, NIST); public-facing transparency summary.

- **Q4:** Year-end **Risk Prioritization Matrix** refresh (**Tool 3**); policy alignment check (HIPAA/GDPR, EU AI Act, NIST); public-facing transparency summary.

##### Run-sheet/Checklist per release (major version or model update)

1. Change description & impact assessment (features, data, autonomy deltas).
2. **Tool 4**: updated data lineage, retention, access roles.
3. **Tool 1**: fairness diffs (pre/post), affected subgroups, mitigation.
4. **Tool 2**: cultural review sign-offs (language, avatars, scenes).
5. **Tool 3**: updated Risk Prioritization Scores; test of hard stops/panic triggers.
6. Re-consent need assessed (material change?).
7. Rollback plan & monitoring metrics defined (alerts, thresholds).
8. MOB approval recorded; go-live window scheduled; owner assigned.

**Table 7:** Who is responsible, accountable, consulting, and informed (RACI)?

Task	Clinician	XR/AI Engineer	Privacy/Law	Ethicist	Patient/Peer	PM
Risk register & Tool 3	A	C	C	R	C	R
Data flow (Tool 4)	C	R	A	C	I	R
Bias audit (Tool 1)	A	R	C	C	I	R
Cultural review (Tool 2)	C	C	C	A	R	R
Consent UX & re-consent	R	C	A	C	C	R
Incident response & drill	A	C	R	C	I	R

(A = Accountable, *R* = Responsible, C = Consult, I = Informed)

For Full Implementation (Tier 1), this table demonstrates who could do what. Responsible (R): does the work. Accountable (A): owns the outcome and signs off (one A per task). There is one A per row by design. Consulted (C): gives expert input before decisions. Informed (I): kept in the loop after decisions/changes. PM = Project Manager (or Program Manager, depending on org): coordinates schedules, agendas, checklists, documentation, and follow-ups; ensures Corrective and Preventive Action (CAPA) items are tracked and closed. For more details, see Appendix C. An organization can mark the “Informed” column with named roles (e.g., “Chief Privacy Officer”) instead of groups.

#### Tier 2—Integrated implementation (leveraging existing committees)

**Who:** Fold ONRAMP into IRB, Clinical Ethics Committee, or Health IT governance. Cadence: Semiannual formal reviews + pre-release checkpoints for major updates.

##### Practical guidelines

- **Gatekeeping form** (2 pages) attached to committee agenda:
  o GTS score + Tier justification,
  o Summary of Tool 4 data flow,
  o Most-recent Tool 1/2/3 results with red-flag items,
  o Human-in-the-loop boundaries (what AI cannot do).
- **Committee checklist** (abbreviated from Tier 1):
  0. Any change to data categories/retention? (Tool 4)
  1. Any subgroup performance regressions? (Tool 1)
  2. Any cultural harms reported or content deltas? (Tool 2)
  3. Are stop-rules, panic triggers, escalation thresholds intact? (Tool 3/Step 4)
  4. Re-consent required? Patient-facing notices updated?
  5. Incident log reviewed; CAPA actions closed?

**Lightweight RACI:** committee chair **A**, service line lead **R**, privacy & clinician **C**.

#### Tier 3—Practitioner-level implementation (lightweight)

- Use Tools 1–4 within supervision/case-conference; document GTS each quarter.
- Pre-deployment mini-checklist (1 page): data map, bias snapshot, cultural rubric, risk matrix; name the supervising clinician who signs off.
- Escalate to Tier 2 if any factor score increases by ≥ 1 point or GTS crosses 8.

##### Continuous refinement, piloting, and escalation

Consistent with best practices in digital health governance, we recommend trial implementation in limited settings, with **Step 6 (Review & Iteration)** guiding refinement. Programs should:

- Start at the lowest safe tier determined by GTS,
- Re-score quarterly and after material changes; escalate tier if risk, autonomy, scale, or sensitivity rises,
- Publish brief, de-identified oversight summaries to bolster trust and invite external feedback.

##### Clinician preparedness and competency

We adapt the Digital Health Competencies in Medical Education (DECODE) framework, an international Delphi consensus with 200+ experts across 79 countries that organizes digital-health competencies into four domains and distinguishes mandatory versus discretionary outcomes, to immersive, AI-enabled psychotherapy [37]. We map these domains to ONRAMP steps/tools so competency expectations are auditable within governance (pre-release checklists, gatekeeping forms, and MOB reviews). Competencies are organized as knowledge, skills, and attitudes (K/S/A), mapped to ONRAMP steps/tools, and designated as Mandatory (all tiers) or Discretionary (primarily Tier 1–2 deployments).

#### Domain A—Professionalism & clinical ethics in digital health

**Focus:** autonomy, consent in adaptive systems, accountability, legal/ethical duties.

**Knowledge (Mandatory):** core bioethics; informed consent in dynamic/AI contexts; documentation standards; high-level liability doctrines (duty to protect/warn; institutional responsibility; FDA SaMD; disclosure requirements).

**Skills (Mandatory):** conduct a layered/interactive consent walkthrough for XR/AI; explain limits of automation and where human-in-the-loop is required; document boundaries of AI autonomy in the care plan.

**Attitudes (Mandatory):** transparency, accountability, non-maleficence; willingness to pause/override automation.

**ONRAMP map: Step 4: Consent & Mitigation Planning**; **Step 5: Implementation & Oversight**; **Tool 4** (VR/AR Data Flow Mapping).

**Discretionary (Tier 1–2):** lead consent UX reviews; contribute to governance policies and escalation thresholds.

##### Domain B—Patient & population digital health 

**Focus:** cultural safety, accessibility, equity, stigma reduction, inclusion.

**Knowledge (Mandatory):** social determinants of mental health; accessibility barriers (cost, bandwidth, space, literacy); cultural considerations in immersive content.

**Skills (Mandatory):** apply **Tool 2: Cultural Sensitivity Rubric** to avatars, narratives, symbols, and language; adapt exposures to patient values; identify equity risks and referral pathways.

**Attitudes (Mandatory):** cultural humility; patient-centeredness; inclusivity.

**ONRAMP map: Step 3: Structured Risk Identification** (equity/culture); **Step 6: Review & Iteration** (equity metrics).

**Discretionary (Tier 1–2):** co-design with community advisors; lead equity audits and remediation plans.

##### Domain C—Health information systems & data governance 

**Focus:** privacy, security, data lineage, regulatory alignment.

**Knowledge (Mandatory):** data classes (PHI/PII, biometric/affective streams), retention/rights (HIPAA/GDPR/CCPA), consent vs. authorization, audit trails, change control.

**Skills (Mandatory):** complete a **Tool 4** data-flow map (collection → processing → storage → access); read access logs; recognize when re-consent is required after material updates.

**Attitudes (Mandatory):** data stewardship; minimum-necessary principle.

**ONRAMP map: Step 1** (declare jurisdiction/stack), **Step 4** (consent & controls), **Step 5** (oversight, audits).

**Discretionary (Tier 1–2):** participate in security/privacy reviews; verify post-market surveillance and CAPA closures.

##### Domain D—Health data science & AI 

**Focus:** AI fundamentals for clinicians; bias/fairness; model drift; “GIGO” data quality.

**Knowledge (Mandatory):** basics of ML workflow; performance metrics (AUROC, sensitivity/specificity, calibration); GIGO risks; sources of bias (sampling, labeling, sensors).

**Skills (Mandatory):** interpret **Tool 1: Biometric Bias Audit** outputs (subgroup performance, disparity ratios); use **Tool 3: Risk Prioritization Matrix** to set/justify harm thresholds and “stop rules”; read model notes/cards for data provenance and update history.

**Attitudes (Mandatory):** healthy skepticism toward automation; avoid automation bias; commit to explainability to patients.

**ONRAMP map: Step 3** (bias/culture/risk identification with Tools 1–3), **Step 6** (drift/equity monitoring).

**Discretionary (Tier 1–2):** participate in pre-release bias reviews; propose mitigation (thresholds, content changes); co-author transparency summaries.

**Table 8:** Minimum competency expectations by tier

Tier	Minimum Competency Coverage	Validation
Tier 3 (Practitioner)	Domain A–D **Mandatory K/S/A**	Supervisor attestation; observed consent walkthrough; one **Tool 1** bias-read exercise; one **Tool 4** data-map exercise.
Tier 2 (Integrated)	All Tier 3 + selected **Discretionary** in A–D	Committee sign-off; case-based objective structured clinical examination (OSCE) (immersion risk + escalation); participation in one equity audit and one release checklist.
Tier 1 (Full)	All above; lead/teach components; governance participation	MOB participation; documented leadership in one release review; authorship of transparency/audit summary.

Mandatory competencies (for all tiers) cover consent/accountability, cultural safety, data governance, and basic AI/bias interpretation, with discretionary competencies emphasized for Tier 1–2 implementations. Competency verification is embedded into pre-release checklists and oversight reviews, creating auditable assurance that clinicians can apply ONRAMP ethically and effectively. This DECODE-aligned framework turns “literacy” into clinically meaningful knowledge, skills, and attitudes (K/S/A) tied to ONRAMP steps and tools, ensuring clinicians can consent, supervise, and troubleshoot AI-immersive therapy responsibly

### Assessment & maintenance (practical and auditable)

- **Assessment methods:** short multiple choice questions/KSA checks; simulated XR consent OSCE; portfolio of one bias audit interpretation (Tool 1), one cultural rubric application (Tool 2), one risk matrix (Tool 3), and one data-flow (Tool 4).
- **Recertification:** annual attestation + a refresher on any system that underwent material change (re-consent review).
- **Link to governance:** competencies are checked in pre-release checklists (Tier 1) or gatekeeping forms (Tier 2); gaps trigger remediation or tier escalation.

## Discussion

While prior literature has called for responsible AI guidelines in psychotherapy [4, 5], the ONRAMP-AI-VRAR protocol represents a novel framework for systematically addressing ethical, legal, and clinical risks in immersive, AI-driven psychotherapy. By embedding structured tools, stakeholder governance, and continuous feedback mechanisms into each stage of the development lifecycle, ONRAMP offers a proactive alternative to retrospective, compliance-only approaches.

### Operationalizing ethical foresight

Traditional bioethical models have struggled to keep pace with the rapid convergence of AI and extended reality (XR) technologies. This protocol reframes ethical governance as a dynamic, iterative process rather than a static checklist. Through tools such as the Biometric Bias Audit Checklist, Risk Prioritization Matrix, and Cultural Sensitivity Rubric, the protocol ensures that AI and immersive content are continuously interrogated for fairness, safety, and inclusivity. The integration of a Multidisciplinary Oversight Board (MOB) operationalizes ethical foresight through structured review cycles, incident response, and performance audits aligned with international calls for anticipatory and participatory AI governance [26, 38].

### Implementation barriers

Real-world adoption will require addressing several practical challenges, including technical complexity, regulatory uncertainty, resource constraints, cost and infrastructure limitations (e.g. hardware, clinician training time) and cultural adaptability. Not all clinics or developers possess the expertise to implement immersive biometric systems or conduct equity audits. Partnerships with academic institutions, open-source tools, and clinician education may help lower this barrier. Additionally, AI-augmented immersive therapies exist at the intersection of medical device regulation, data privacy law, and professional licensure. While ONRAMP-AI-VRAR provides regulatory scaffolding (e.g., alignment with HIPAA, GDPR, and FDA SaMD guidance), cross-jurisdictional consistency remains limited. Many mental health settings, particularly in low-resource or community environments, lack the funding and technical infrastructure to support immersive therapy deployment, let alone oversight boards or dynamic consent systems. Moreover, global implementation will require adapting ONRAMP tools (e.g., the Cultural Sensitivity Rubric) to local norms, languages, and clinical practices. Community partnerships and participatory design processes are essential to avoid digital colonialism and ensure contextual relevance.

### Implications for future research and policy

The ONRAMP framework invites new directions in both empirical research and policy development:

- Clinical trials should incorporate ONRAMP tools as part of study protocols, documenting ethical safeguards alongside efficacy and safety outcomes.
- Standardization bodies such as the FDA, WHO, and the International Organization for Standardization (ISO) could consider incorporating elements of ONRAMP into guidelines for therapeutic XR systems and AI SaMD (Software as a Medical Device).
- Health equity research should evaluate whether ONRAMP-based deployments improve access, reduce bias, or promote trust in marginalized populations. Moreover, cross-cultural validation of the Cultural Sensitivity Rubric is essential.
- AI explainability and consent science may benefit from ONRAMP’s layered, immersive-informed consent approaches, particularly in populations with limited health literacy.
- Participatory ethics should be embedded in future iterations, expanding the role of patients, peer specialists, and community advocates in co-design and governance.

## Limitations

A key limitation of the present work is that the four ONRAMP tools should be understood as conceptual prototypes rather than validated instruments. Their current form relies on subjective rating scales (e.g., 1–5 ratings, low/medium/high impact), which poses risks to inter-rater reliability. Without defined anchors for each rating point, two reviewers may generate inconsistent results. Likewise, the Risk Prioritization Matrix multiplies Likelihood and Impact scores, implicitly assuming an interval-level relationship that has not been empirically demonstrated. This raises concerns regarding both construct and content validity. Additionally, this work synthesizes ethics/governance concepts via a targeted narrative scan rather than a comprehensive systematic review; we did not perform formal risk-of-bias or certainty appraisals, and findings should be interpreted as guidance for operational governance rather than effect-size estimates. Future work must provide operational definitions for each rating category, establish scoring anchors, and conduct psychometric testing (e.g., inter-rater reliability, factor structure, predictive validity) before clinical or research deployment. Until such validation is complete, the ONRAMP tools should be treated as proof-of-concept frameworks designed to stimulate discussion and guide early implementation, not as definitive measurement instruments.

## Conclusion

The ONRAMP-AI-VRAR protocol provides a concrete, operational framework for managing the distinctive “Garbage In, Garbage Out” (GIGO) risks inherent to AI-driven immersive psychotherapy, particularly those arising from biased training data, unvalidated adaptive content, and opaque data pipelines. By embedding upstream audits, participatory design tools, and dynamic oversight mechanisms, the protocol translates ethical principles into enforceable safeguards tailored to the unique characteristics of immersive environments, such as avatar realism, real-time affective feedback, and user vulnerability during heightened states of presence.

A key contribution of ONRAMP-AI-VRAR is its explicit engagement with the GIGO problem: when poor-quality or biased biometric inputs, like facial expressions, motion patterns, or vocal cues, lead to inaccurate classification, maladaptive therapeutic responses, or unintended psychological harm. The protocol addresses these risks through a structured process of system mapping, risk anticipation, consent design, and multidisciplinary oversight both prior to deployment and through continuous lifecycle monitoring. Future work should assess its application across diverse clinical populations and technology platforms.

## Electronic supplementary material

Below is the link to the electronic supplementary material.

Supplementary Material 1

Supplementary Material 2

## Data Availability

All data generated or analysed during this study are included in this published article and its supplementary information files. This work reports a targeted, narrative evidence scan to support the ONRAMP-AI-VR/AR governance framework; sources are cited throughout, and no new datasets were created. Supplementary materials include the PRISMA 2020 checklist and flow diagram and the four ONRAMP implementation tools (Biometric Bias Audit Checklist, Cultural Sensitivity Rubric, Immersion-Specific Risk Severity Matrix, and VR/AR Data Flow Mapping Template).

## Appendix A: Full tool templates

**Tool 1: Biometric bias audit checklist**

**Purpose**: Evaluate whether biometric inputs (e.g., heart rate, pupil dilation, facial emotion recognition) are trained, tested, and validated across diverse populations to minimize algorithmic bias

**Table:**

Checklist Item	Yes/No	Notes/Action Plan
Are training datasets demographically representative (race, age, gender, etc.)?		
Have synthetic or oversampled data been used to address underrepresentation?		
Are physiological variations (e.g., skin tone, heart rate variability) accounted for in model calibration?		
Have performance disparities been tested across demographic groups?		
Have third-party audits or fairness metrics been applied?		
Is there a process for updating models based on emerging disparity data?		

**Tool 2: Cultural sensitivity rubric for immersive therapeutic content**

**Purpose**: Assess exposure scenarios and virtual environments for cultural appropriateness and inclusivity

**Table:**

Domain	Assessment Question	Rating (1–5)	Notes/Required Modifications
Representation	Are avatars and scenarios reflective of diverse racial, ethnic, and gender identities?		
Trauma Sensitivity	Are traumatic content elements adapted to the user’s cultural background and lived experience?		
Language	Are voiceovers, instructions, and dialog available in the user’s primary language(s)?		
Symbolism	Are potentially harmful or stigmatizing symbols/images avoided or explained?		
Religious/Cultural Context	Are religious/cultural practices or taboos respected (e.g., in dress, interpersonal touch)?		
Review Process	Has the content been reviewed by a culturally diverse panel including patient advocates?		

**Tool 3: Risk prioritization matrix**

**Purpose:** Prioritize mitigation strategies by evaluating the likelihood and impact of immersion-related harms

**Table:**

Risk	Likelihood (Low/Med/High)	Impact (Low/Med/High)	Risk Prioritization Score (1–9)	Mitigation Strategy
Simulator sickness (nausea, dizziness)				
Dissociation or derealization				
Re-traumatization (PTSD exposure therapy)				
Overexposure to phobic stimuli				
Spatial privacy violation (AR mapping of real spaces)				
Emotional blunting from overuse				

Risk Prioritization Score = Likelihood ×Impact (1–3 scale each; max 9). This is an ordinal ranking for mitigation priority (not a clinical measurement)

**Tool 4: VR/AR data flow mapping template**

**Purpose**: Clarify how user data is collected, stored, processed, and shared within immersive therapeutic systems. This tool can be used to inform consent language, guide privacy audits, and satisfy GDPR/CCPA/ONC compliance

**Table:**

Data Type	Source	Collection Method	Use in AI Decision-Making	Storage Location	Access Rights	Retention Period
Facial expressions	Camera	Real-time image analysis	Emotion detection for adaptive feedback	Cloud (provider like AWS)	Developer, therapist	30 days
Heart rate	Wearable sensor	Continuous streaming	Used to adjust exposure intensity	Local device	Therapist only	Session only
Voice recordings	Microphone	Session logging	Transcribed and sentiment analyzed	Encrypted cloud	Developer (QA), therapist	6 months
Movement patterns	Motion tracking	Spatial data collection	Exposure sequencing	Cloud	Developer	90 days

## Appendix B: Detailed risk identification and mitigation table

**Table:**

Risk Category	Specific Risk	ONRAMP Step(s)	Tool/Protocol Element	Tailored Mitigation Strategy
Psychological Harm	Overexposure to anxiety-inducing stimuli (e.g., PTSD, OCD)	Step 3: Structured Risk Identification Step 5: Implementation & Oversight	Tool 3: Risk Prioritization Matrix	Real-time biometric stress monitoring, patient-controlled stop mechanisms, therapist dashboard alerts, MOB review of high-risk sessions
Psychological Harm	Dissociation or derealization from immersive sensory overload	Step 3: Structured Risk Identification Step 5: Implementation & Oversight	Tool 3: Risk Prioritization Matrix	Pre-session screening for dissociative risk, restricted session length, intensity modulation options, customizable pacing
Privacy and Surveillance	Biometric or spatial data exposure (e.g., gaze, facial, room mapping)	Step 1: Scoping & Orientation Step 4: Consent & Mitigation Planning	Tool 4: VR/AR Data Flow Mapping Template	Full data flow audit, access rights review, data minimization and encryption, compliance checks with HIPAA/GDPR/ONC
Bias and Discrimination	Algorithmic bias in emotion/behavioral recognition across demographics	Step 2: Gap & Needs Assessment Step 3: Structured Risk Identification Step 4: Consent & Mitigation Planning	Tool 1: Biometric Bias Audit Checklist	Third-party fairness audits, testing on synthetic diverse cohorts, retraining on representative datasets, transparency reports
Cultural Incongruence	Exposure scenarios reinforce stereotypes or misalign with cultural norms	Step 3: Structured Risk Identification	Tool 2: Cultural Sensitivity Rubric	Cultural panel review, rejection of scenarios scoring < 3, user-adjustable content filters, quarterly oversight board review
Consent and Comprehension	Inadequate understanding of therapy risks or AI functions	Step 1: Scoping & Orientation Step 5: Implementation & Oversight	Tool 4: VR/AR Data Flow Mapping Template Interactive Consent (ONRAMP Step 1 & 5)	VR-based demo consent sessions, layered and readable disclosures, re-consent after updates, pre-therapy orientation
Overreliance on Automation	AI makes unsupervised therapeutic decisions beyond its intended scope	Step 4: Consent & Mitigation Planning Step 5: Implementation & Oversight	ONRAMP Oversight Governance Therapist Dashboard	Hard-coded AI boundaries, therapist override triggers, required post-session review for all AI-driven decisions
Equity and Access	Disparities due to cost, digital literacy, or broadband access	Step 1: Scoping & Orientation Step 5: Implementation & Oversight	Addressed through ONRAMP governance and design philosophy	Mobile-compatible, low-bandwidth versions, Medicaid/public partnerships, demographic outreach audits, device subsidies

## Appendix C. CAPA log template (corrective and preventive action)

Use this template to document adverse events or non-conformities, root causes, corrective and preventive actions, verification of effectiveness, and closure. Link each CAPA to ONRAMP-AI-VRAR steps/tools and the governance tier at the time of the event.

**Table:**

Project/Protocol	Version/Build	Date Range	Owner (Accountable)
-	-	-	-

## CAPA register (summary log)

**Table:**

CAPA ID	Date Opened	Source (incident/audit/UX)	Severity (L/M/H)	Summary of Issue	Owner (R)	Accountable (A)	Due Date	Status
-	-	-	-	-	-	-	-	-
-	-	-	-	-	-	-	-	-
-	-	-	-	-	-	-	-	-
-	-	-	-	-	-	-	-	-
-	-	-	-	-	-	-	-	-
-	-	-	-	-	-	-	-	-
-	-	-	-	-	-	-	-	-
-	-	-	-	-	-	-	-	-

Note: Severity refers to potential patient impact before mitigation; status options include Open/In Progress/Verified/Closed

## C.2 CAPA record (single item)

**Table:**

CAPA ID
Date Opened
Linked ONRAMP Elements (Steps/Tools)
Governance Tier & GTS at Event (Tier, Score)
Affected Versions (Model/Content/App)
Description of Non-Conformity/Issue
Root Cause(s) (e.g., 5 Whys/fishbone)
Corrective Action(s) (what to fix now)
Preventive Action(s) (how to prevent recurrence)
Owner(s) & Due Date(s)
Verification of Effectiveness (method, metric, date, result)
Closure (date) & Sign‑offs (A/QA/MOB)

RACI Legend & Glossary

*R* = Responsible; A = Accountable; C = Consulted; I = Informed. PM = Project/Program Manager. CAPA = Corrective and Preventive Action

Discovery & Re‑consent: indicate if material system changes require re‑consent; attach patient‑facing notices if applicable
