# Supplementary material for: ONRAMP-AI-VRAR: an operational protocol for ethics and governance of AI-enabled immersive psychotherapy
Source: BMC Psychiatry. 2025 Dec 11;25:1145. doi: 10.1186/s12888-025-07557-x (PMC12696944; doi:10.1186/s12888-025-07557-x)
Supplement: Supplementary file 2 — Supplementary Material 2 [file 12888_2025_7557_MOESM2_ESM.docx]

**Identification of studies via databases and registers**

Records removed *before screening*:

Duplicate records removed (n = 0)

Records marked as ineligible by automation tools (n = 0)

Records removed for other reasons (n = 0)

Records identified from*:

Databases (n = 3)

Registers (n = 1)

**Identification**

Records screened

(n = 913)

Records excluded**

(n = 870)

Reports sought for retrieval

(n = 43)

Reports not retrieved

(n = 0)

**Screening**

Reports assessed for eligibility

(n = 43)

Reports excluded:

Not psychotherapy (n = 9)

Engineering-only without clinical application (n = 11)

No ethics/governance content (n = 8)

etc.

Studies included in review

(n = 15)

Reports of included studies

(n = 15)

**Included**

Note: Following peer review, 3 conceptually relevant works were incorporated into the narrative synthesis (total discussed = 18), but only studies meeting a priori inclusion criteria are counted as 'included' in the PRISMA flow.

Source: Page MJ, et al. BMJ 2021;372:n71. doi: 10.1136/bmj.n71.

This work is licensed under CC BY 4.0. To view a copy of this license, visit <https://creativecommons.org/licenses/by/4.0/>
